# Diurnal Effects on the Fraction of Fetal Cell‐Free DNA in Maternal Plasma

**DOI:** 10.1002/pd.6836

**Published:** 2025-06-18

**Authors:** Alexander Gamisch, Julia Hess, Maria‐Elisabeth Mustafa‐Korninger

**Affiliations:** ^1^ Medilab Dr. Mustafa Dr. Richter Labor für Medizinisch‐chemische und Mikrobiologische Diagnostik GmbH Molekularbiologie Salzburg Austria

## Abstract

**Objective:**

The discovery of fetal cell‐free DNA (cfDNA) has revolutionized prenatal diagnostics through non‐invasive prenatal testing (NIPT), which depends on accurately measuring the fetal fraction (FF) in maternal plasma. While FF is known to be influenced by maternal and fetal factors, the impact of intraday rhythms remains unclear. This study investigated whether FF varies based on blood draw timing.

**Methods:**

Data from 2519 euploid singleton pregnancies undergoing NIPT were analyzed. Key variables included maternal age, BMI, gestational age, fetal sex, and blood draw timing (06:50–21:00). FF was measured using the Harmony Prenatal IVD Test. Multiple linear regression identified independent predictors of FF, while intraday variation was assessed using Mann‐Whitney U tests and boxplots.

**Results:**

FF showed a significant positive relationship with blood draw timing (*β* = 0.00176 per hour, *p* < 0.005), with afternoon values approximately 10% higher than morning values (∼0.01 difference). Other predictors included BMI (negative), gestational age (positive), and fetal sex (higher in females). Blood draw timing appeared to be a stronger predictor of FF than gestational age or fetal sex, second only to BMI.

**Conclusion:**

This novel finding demonstrates diurnal variation in FF, suggesting that optimizing blood draw timing could improve NIPT accuracy, particularly in borderline cases. Further research is needed to confirm the clinical implications.

**Summary**

- What's already known about this topic?
  ◦ Fetal fraction (FF), a critical parameter for prenatal diagnostics through non‐invasive prenatal testing (NIPT), is influenced by maternal and fetal physiological factors; however, the potential impact of intraday rhythms on FF remains poorly understood.
- What does this study add?
  ◦ FF demonstrates significant diurnal variation, with higher values in the afternoon. This novel finding highlights the importance of timing in FF measurement and suggests optimizing blood draw timing could improve NIPT accuracy, particularly in borderline cases.

## 1 Introduction

Non‐invasive prenatal testing (NIPT) has revolutionized prenatal care by enabling early and accurate detection of fetal chromosomal abnormalities (such as trisomy 13, 18, and 21) through the analysis of cell‐free DNA (cfDNA) in maternal plasma [1, 2, 3]. cfDNA in maternal blood comprises a mix of small genomic DNA fragments (< 200 base pairs) released primarily from the mother's hematopoietic system and organs, while the smaller fetal DNA fraction is derived mainly from syncytiotrophoblast cell turnover in the placenta [3, 4, 5, 6]. Fetal fraction (FF), defined as the ratio of fetal cfDNA to total circulating cfDNA in maternal plasma, is a critical parameter for ensuring NIPT accuracy and interpreting clinical outcomes [5, 6]. Estimates of FF can vary significantly between and within pregnancies but generally range between 0.10 and 0.15 during gestational weeks 10–20 (the most common period for NIPT) [5, 6, 7].

In general, higher FFs allow greater statistical separation between aneuploid and euploid pregnancies, enhancing test performance. Conversely, low FFs reduce detection rates and may lead to uninformative or “no call” outcomes if the FF does not meet the assay‐specific minimum threshold, typically ranging from 0.02 to 0.04 [5, 7, 8, 9, 10]. Although most NIPT tests yield successful results, approximately 1%–8% of pregnant women receive uninformative or no‐call outcomes, primarily due to insufficient FF [5, 7, 9]. Such cases pose significant challenges for clinical management, as they often necessitate either a repeat test using a second blood sample or invasive procedures such as amniocentesis, which carry additional risks to maternal and fetal health [1, 2]. Consequently, identifying and understanding factors influencing FF has been a central focus to enhance test performance and reduce the rate of screening failures [1, 6, 11, 12].

In theory, FF is determined by the balance between maternal and fetal cfDNA levels in maternal plasma [9, 12]. Thus, any factor affecting the concentrations of maternal or placental circulating cfDNA can influence FF. For instance, maternal circulating cfDNA levels may increase or placental cfDNA levels may decrease due to various biological processes [6, 12]. While many aspects of cfDNA biology remain largely unknown, some factors have already been identified to influence the release of cfDNA via apoptosis, necrosis, and/or active secretion as possible mechanisms (e.g., exercise, inflammatory processes, surgeries, therapies, and lifestyle factors) [6, 13, 14, 15]. Additionally, physiological variables, including gestational age, maternal BMI, and fetal sex, are known to influence FF [1, 5, 6, 7].

Emerging evidence suggests that circadian or intraday rhythms may also influence circulating cfDNA levels [13, 14, 16, 17, 18] (but see Refs. [19, 20, 21]). However, the effect of these temporal patterns on FF in maternal blood remains poorly understood. Illuminating this potential influence is critical, as it could enhance test performance by optimizing blood collection timing, particularly in borderline cases where FF approaches the assay's sensitivity threshold. To the best of our knowledge, no studies have directly examined how FF levels vary with sampling times throughout the day. This retrospective study aimed to investigate whether a relationship exists between FF levels and the time of blood sample collection and to evaluate the robustness of these findings when considering outliers, gestational age, BMI, and fetal sex.

## 2 Material and Methods

### 2.1 Study Population

A retrospective study was conducted on samples from women undergoing NIPT testing at Medilab GmbH, Salzburg. Between June 2023 and October 2024, NIPT was performed on women from the regional (predominantly Central European) population of Salzburg, Austria. Data on the date and time of blood draw, maternal age, gestational age, and body mass index (BMI) at the time of sampling were collected by the ordering physician. Additionally, fetal sex (male or female) and the proportion of fetal cfDNA, referred to as FF, calculated by the NIPT test were recorded. An FF of 0.1 indicates that 10% of the cfDNA in maternal plasma is of fetal origin. Multiple pregnancies and samples with missing data (e.g., BMI, time of blood draw) were excluded from the dataset. Similarly, samples that failed NIPT analysis as well as those with a NIPT probability > 0.01 for sex chromosome aneuploidies (*n* = 6), trisomy 13 (*n* = 4), trisomy 18 (*n* = 4), or trisomy 21 (*n* = 10) were excluded. The final dataset included 2519 unique “euploid” singleton pregnancies (*n*_male_ = 1050, *n*_female_ = 993, *n*_NA_ = 476) with blood draws conducted between 06:50 and 21:00 (see Figure S1A–E, Table S1). The study was conducted in accordance with the Declaration of Helsinki. All participants provided written informed consent to undergo NIPT and to allow their anonymized results and data to be used in scientific research.

### 2.2 Sample Preparation and NIPT

Peripheral blood samples (≥ 10 + 0 weeks' gestation) were collected offsite in Cell‐Free DNA Collection Tubes (Roche Austria GmbH, Vienna, Austria) and stored at room temperature until plasma separation. Plasma separation, cfDNA extraction, and NIPT analysis were completed within 7 days. cfDNA was extracted using the QIA Symphony SP/AS Platform (Qiagen, Hilden, Germany) and analyzed with the microarray‐based Harmony Prenatal IVD Test (Roche Austria GmbH, Vienna, Austria) at Medilab GmbH Salzburg.

The Harmony Prenatal Test is a cfDNA‐based screening test for trisomies 13, 18, and 21 [22]. It targets specific chromosomes using ∼6800 Digital Analysis of Selected Regions (DANSR) assays, which analyze polymorphic and non‐polymorphic loci to estimate chromosome proportions and fetal fraction [23]. The Fetal‐fraction Optimized Risk of Trisomy Evaluation (FORTE) algorithm processes DANSR data to quantify chromosomes 4, 13, 18, 21, and X, integrating fetal fraction estimates with maternal and gestational age to calculate individualized trisomy risk scores [22]. The test requires a minimum fetal fraction of 0.04 for valid results [7].

### 2.3 Statistical Analysis

To investigate the potential intraday effect on FF, we employed several complementary approaches. First, a multiple linear regression analysis was conducted to disentangle the effects of independent variables (i.e., maternal BMI, maternal age, gestational age, fetal sex, and clock time of blood draw) on the dependent variable (FF) while keeping all other variables constant. For this analysis, the clock time of blood draw was converted to a continuous numeric format (e.g., 07:30 = 7.5), and fetal sex was converted to a categorical variable (1 = Male, 0 = Female, 2 = undetermined). Model significance was assessed using an F‐test, and the statistical significance of individual predictors was evaluated using *t*‐tests and corresponding *p*‐values. To ensure robustness against potential violations of linear regression assumptions, such as the presence of outliers, non‐normality of residuals, or heteroscedasticity, robust multiple linear regression was also performed. *p*‐values for robust regression were approximated by comparing robust *t*‐statistics to critical values from the *t*‐distribution. Standardized regression coefficients (SRCs) were calculated to assess the relative importance of each predictor to the model. By standardizing predictors and the outcome variable, SRCs allow direct comparison of effect sizes, reflecting the change in FF per one‐standard‐deviation change in each predictor. To further explore intraday variations in FF, we divided the dataset by clock hour of blood draw and visualized the distributions via boxplots. We then conducted an exploratory series of pairwise non‐parametric Mann‐Whitney U tests for each combination of clock hours. For this purpose, hours with low sample sizes (6:00, 20:00, and 21:00, with *n* = 2, 7, and 1, respectively) were excluded from the analysis. Additionally, to examine a broader categorical intraday effect and ensure that the observed diurnal variation in FF was not solely dependent on a specific cutoff time, we divided the dataset into three sets (A–C), each using slightly different thresholds to define “morning” and “afternoon” populations, based on the results of pairwise Mann–Whitney U tests. Specifically, for set A, blood draws between 06:50 and 12:00 were assigned to the “morning” population, and those between 12:01 and 21:00 to the “afternoon” population. Sets B and C were constructed similarly using the ranges 06:50–13:00 & 13:01–21:00 and 06:50–14:00 & 14:01–21:00, respectively. Density plots were generated to visualize FF distributions, and Mann‐Whitney U tests were used to compare FF values between the populations in each set. Additionally, Mann‐Whitney U tests were conducted to compare the distributions of BMI and gestational age between the morning and afternoon populations in all three sets. These tests evaluated whether any differences in FF could be attributed to unequal group composition with respect to these covariates. To quantify effect sizes, the rank‐biserial correlation was calculated. This metric ranges from +1 (complete separation) to −1 (complete inverse separation) with 0 indicating complete overlap. Positive values suggest that one group generally has higher values, while negative values suggest the opposite. The robustness of morning versus afternoon comparisons was further explored in subsamples based on fetal sex (male, female), gestational age (< 105 days, approximately week 15), BMI (< 24.9, 25–29.9, > 30) and with IVF pregnancies excluded. A sensitivity analysis was conducted by repeating the analysis with extreme FF values (FF > 0.1 and < 0.2) excluded to determine whether the original results were driven by outliers. Descriptive FF statistics (the mean, median) were computed for each of the populations and the delta mean FF (meanFF_afternoon_ − meanFF_morning_) was calculated. All statistical analyses were performed using R v. 4.4.1 [24] with the functionalities of the packages *dplyr* [25], QuantPsyc [26], *ggplot2* [27], and *MASS* [28]. A *p*‐value of < 0.05 was used as the threshold for significance.

## 3 Results

### 3.1 Study Population Characteristics

The basic pregnancy characteristics of the study population of 2519 pregnant women, including maternal age, body mass index (BMI), gestational age, and FF, are presented in Table 1 (see also Figure S1A–E, Table S1). The study cohort had a mean gestational age of 89.69 days (∼13 weeks; IQR: 83–94 days), a mean maternal age of 34.18 years (IQR: 31–37), and a mean BMI of 24.28 kg/m^2^ (IQR: 21.04–26.45). The mean fetal fraction was 0.12 (IQR: 0.09–0.14) and the median clock hour of blood draw was 12:00 (IQR: 10:00–14:45). Most pregnancies (94%, 2367/2519) were between 10 and 15 weeks of gestation, and 61% (1538/2519) of pregnant women were ≤ 35 years old. A total of 246 pregnancies were derived through in vitro fertilization (IVF). BMI distribution included 67 women with BMI < 18.5, 1553 with BMI 18.5–24.9, 569 with BMI 25–29.9, 204 with BMI 30–34.9, and 88 with BMI > 35.

**TABLE 1:** Basic characteristics of the study population.

Variable	Mean	Median	25th percentile	75th percentile	Minimum	Maximum
Gestational age (days)	89.69	88	83	94	70	260
Maternal age (years)	34.18	34	31	37	18	55
Maternal BMI (kg/m^2^)	24.28	23.18	21.04	26.45	15.7	48.05
Fetal fraction (FF)	0.12	0.11	0.09	0.14	0.04	0.34
Time of blood draw (decimal hours)	12.54	12	10	14.75	6.83	21

### 3.2 Multiple Regression Analyses

A multiple linear regression analysis was performed to examine the factors associated with FF, including maternal BMI, maternal age, gestational age, clock hour of blood draw, and fetal sex (Table 2). The linear regression model examining the predictors of FF was statistically significant overall, with an F (6, 2512) = 86.04, *p* < 2.2 × 10^−16^. The model explained approximately 17.05% of the variance in FF (*R* ^2^ = 0.1705), with a slightly adjusted *R* ^2^ = 0.1685 accounting for the number of predictors. This suggests the presence of other unmeasured factors influencing FF. Statistically significant predictors (*p* < 0.005) included maternal BMI, gestational age, fetal sex, and clock hour of blood draw, while maternal age did not have a significant impact on FF (Table 2). The standardized regression coefficients (SRCs) revealed that BMI (SRC = −0.376) had the highest contribution to the linear model followed by the clock hour of blood draw (SRC = 0.139), gestational age (SRC = 0.110), and fetal sex (SRC = −0.062). Specifically, the regression coefficients (*β*) for each predictor, while holding other variables constant, indicated a significant negative relationship between BMI and FF, with FF decreasing by 0.00329 for every one‐unit increase in maternal BMI. Conversely, there was a significant positive relationship between gestational age and FF, with FF increasing by 0.00033 for each additional day of gestation. Female fetuses were associated with a 0.00337 unit increase in FF compared with male fetuses. Additionally, the clock hour of blood draw demonstrated a significant positive relationship with FF, showing an increase of 0.00176 per hour between 06:50 and 21:00 (Table 2). The robust multiple linear regression results closely mirrored those of the standard multiple linear regression, confirming that the findings are robust against potential outliers and violations of normality or homoscedasticity assumptions (Table 2).

**TABLE 2:** Results of the multiple regression analysis examining the effects of maternal BMI, age, gestational age, fetal sex, and blood draw time on fetal fraction (FF).

Multiple linear regression	Coefficient (*β*)	SRC	Std. error	*t*‐value	*p*‐value
Maternal BMI (kg/m^2^)	−0.00329	−0.37590	1.60E−04	−20.548	**7.52E−87**
Maternal age (years)	0.00001	0.00166	1.52E−04	0.091	0.927
Gestational age (days)	0.00033	0.11041	5.49E−05	5.985	**2.47E−09**
Time of blood draw (decimal hours)	0.00176	0.13863	2.32E−04	7.615	**3.71E−14**
Fetal sex^a^	−0.00337	−0.04175	1.61E−03	−2.095	**3.63E−02**

Robust multiple linear regression	Coefficient (*β*)	SRC	Std. error	*t*‐value	*p*‐value
Maternal BMI (kg/m^2^)	−0.00318	−0.36294	1.57E−04	−20.248	**0.00E+00**
Maternal age (years)	0.00005	0.00630	1.49E−04	0.353	0.724
Gestational age (days)	0.00030	0.10176	5.38E−05	5.630	**2.00E−08**
Time of blood draw (decimal hours)	0.00166	0.13055	2.27E−04	7.319	**3.34E−13**
Fetal sex^a^	−0.00276	−0.03422	1.57E−03	−1.752	0.080

*Note: p*‐values below 0.05 are bold.

Abbreviation: SRC, standardized regression coefficient.

^a^ Male versus female fetuses (estimates for fetuses of unknown sex not shown).

### 3.3 Intraday Variations of FF Based on Clock Time of Blood Draw

**FIGURE 1:**
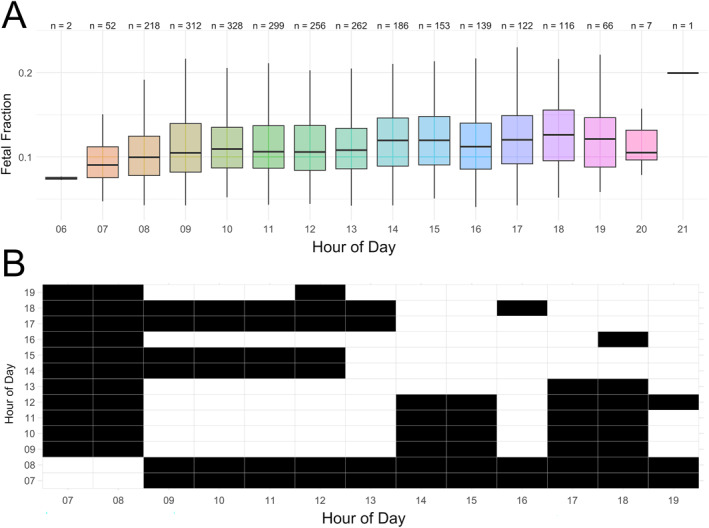
Intraday variations of FF. (A) FF boxplot per hour of blood draw with *n* showing the number of samples, respectively. (B) Result matrix of the series of pairwise Mann‐Whitney U tests (FF). Black cells indicate significant different comparisons (*p*‐value below 0.05).

Intraday variations of FF were further explored via hourly boxplots and a series of pairwise non‐parametric Mann‐Whitney U tests. Visual inspection of the hourly FF boxplots revealed broadly scattered and largely overlapping FF values throughout the day, however, with a subtle trend toward lower FFs in the morning (e.g., from 7:00 to 13:00) compared to the afternoon (e.g., from 14:00 to 19:00; see Figure 1A). Indeed, when we conducted hourly pairwise Mann‐Whitney U tests, we found that the FFs for morning hours were mostly significantly (*p* < 0.05) different than those from the afternoon (Figure 1B). In detail, the FF of clock hours 7 and 8 are significantly different compared to clock hours of the rest of the day. Likewise, the FFs of clock hours 8, 10, 11 and 12 are each significantly different from clock hours 14, 15, 17, 18 and 19 (in case of 12) while being not significantly different from earlier hours (except 7 and 8). Likewise, the FF of clock hours of the afternoon (i.e., 14–19) mostly differ significantly from earlier hours (except for 16) but not from afternoon hours. In summary, these analyses hint at a significant difference in FF between morning and afternoon samples with a possible breaking point around 13:00–14:00.

### 3.4 Relationship Between FF and Clock Time of Blood Draw Between Morning and Afternoon Populations

**FIGURE 2:**
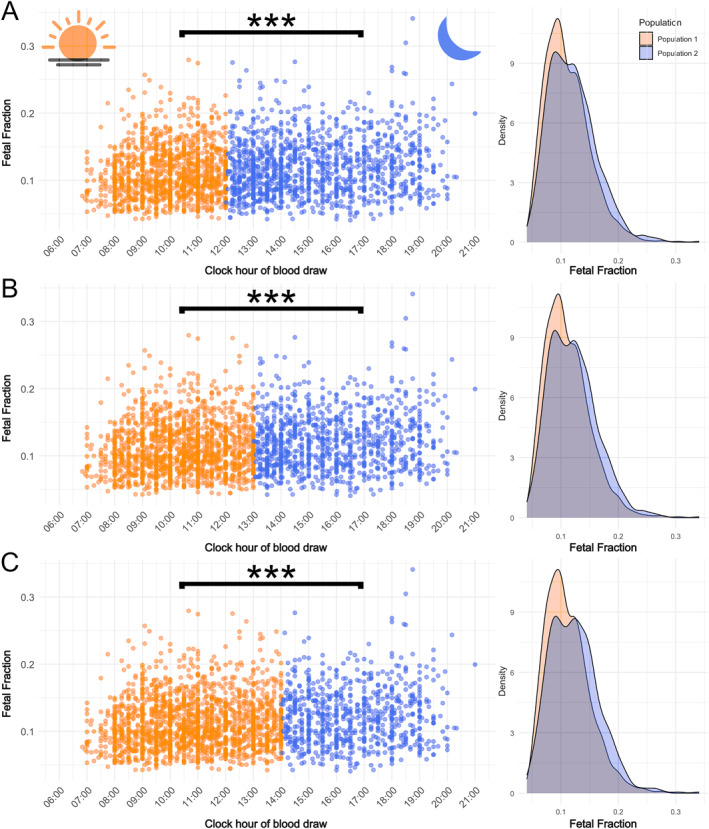
Relationship between FF and clock time of blood draw between three sets of morning (Population 1) and afternoon (Population 2) populations. (A) Set A: 06:50 and 12:00 & 12:01 and 21:00; (B) set B: 06:50–13:00 & 13:01–21:00; (C) set C: 06:50–14:00 & 14:01–21:00. Brackets with asterisks indicate a highly significant difference (*p* < 0.00001) in FF between morning and afternoon populations as found by the Mann‐Whitney U tests (see also Table 3). Corresponding density plots indicate, while largely overlapping, a greater relative concentration of lower FF values in the morning and a greater relative concentration of higher FF values in the afternoon populations, respectively. Samples belonging to the morning population are indicated in orange, while samples belonging to the afternoon population are blue.

To further investigate a potential broad categorical intraday effect on FF we analyzed three different sets of morning and afternoon populations. Overall, the density plots of FF of the three sets of morning and afternoon populations show a similar and slightly bimodal, largely overlapping distribution of FF values (Figure 2A–C). Nevertheless, the morning population has a higher density of lower FF values while the afternoon population has a higher density of higher FF values (Figure 2A–C). In concordance, the Mann‐Whitney U tests and Rank‐Biserial correlation indicated a small but significant difference in FF between the two groups (*p* < 0.001; Table 3). Indeed, on average, the morning populations had c. 1% lower fetal cfDNA percentages than the afternoon populations (i.e., 11.07%–11.18% vs. 11.95% and 12.3%; Table 3). In other words, the FF of the morning populations has c. 8%–10% lower FF values than the afternoon population. This overall pattern was most pronounced in set C (06:50–14:00 & 14:01–21:00) across all comparisons and essentially robust across pregnancies with male and female fetuses, gestational age (< 105 days∼week 15), and after the exclusion of extreme values (FF > 0.1 & < 0.2), IVF pregnancies and to a lesser extent across different BMIs (Table 3). In the latter group, the overall trend of lower FF values in the morning population was consistent across different BMI categories (< 24.9, 25–29.9, > 30). However, the trend was less pronounced and non‐significant in patients with a high BMI (> 30), likely due to the smaller sample size (*n* = 292) and confounding from significantly different gestational ages (Table 3). In contrast to FF, Mann‐Whitney *U* test results comparing gestational age and BMI between morning and afternoon populations were largely non‐significant, except for BMI in Set A of the full dataset and the IVF excluded dataset as well as gestational age in Sets A to C within the BMI > 30 subgroup (Table 3). These findings suggest that the observed differences in FF were unlikely to be driven by imbalances in BMI or gestational age distributions between the morning and afternoon populations.

**TABLE 3:** Descriptive statistics and Mann‐Whitney *U* test results comparing fetal fraction (FF), gestational age (GA) and BMI between “morning” (Population 1) and “afternoon” (Population 2) groups across three different time range definitions (Set A–C).

Dataset	Set	Pop.1 (*n*)	Pop.2 (*n*)	Mean FF_Pop.1_	Mean FF_Pop.2_	Δ (Mean FF_Pop.2_ − mean FF_Pop.1_)	Median FF_Pop.1_	Median FF_Pop.2_	Rank‐biserial corr. (FF)	*p*‐value (FF)	*p*‐value (GA)	*p*‐value (BMI)
Full dataset	A	1280	1239	0.1107	0.1195	0.0088	0.104	0.1154	−0.062	**6.42E−08**	0.125	**0.0364**
Full dataset	B	1541	978	0.1112	0.1211	0.0099	0.1045	0.1178	−0.07	**3.74E−09**	0.427	0.32
Full dataset	C	1779	740	0.1118	0.1229	0.0111	0.1052	0.1199	−0.079	**4.83E−10**	0.251	0.275
IVF excluded	A	1151	1122	0.1111	0.1196	0.0085	0.1042	0.1159	−0.061	**5.56E−07**	0.447	**0.0296**
IVF excluded	B	1379	894	0.1113	0.1215	0.0102	0.1046	0.1182	−0.072	**6.69E−09**	0.173	0.654
IVF excluded	C	1596	677	0.112	0.1231	0.0111	0.1055	0.1204	−0.078	**3.33E−09**	0.128	0.468
Male fetus	A	520	530	0.1107	0.1194	0.0087	0.1053	0.1156	−0.06	**7.32E−04**	0.243	0.403
Male fetus	B	633	417	0.1114	0.1207	0.0093	0.1064	0.1169	−0.062	**6.34E−04**	0.501	0.9
Male fetus	C	724	326	0.1114	0.1233	0.0119	0.106	0.1197	−0.081	**2.64E−05**	0.795	0.169
Female fetus	A	516	477	0.1126	0.1214	0.0088	0.1062	0.117	−0.063	**5.78E−04**	0.388	0.106
Female fetus	B	627	366	0.1134	0.1226	0.0092	0.1067	0.1194	−0.069	**2.99E−04**	0.524	0.763
Female fetus	C	726	267	0.1135	0.1258	0.0123	0.1076	0.1209	−0.088	**2.14E−05**	0.167	0.982
BMI < 24.9	A	805	827	0.1199	0.1288	0.0089	0.1161	0.1248	−0.063	**1.23E−05**	0.843	0.817
BMI < 24.9	B	990	642	0.1202	0.131	0.0108	0.1163	0.1271	−0.077	**1.20E−07**	0.988	0.957
BMI < 24.9	C	1142	490	0.1207	0.1331	0.0124	0.1169	0.1295	−0.089	**1.07E−08**	0.611	0.914
BMI 25–29.9	A	303	266	0.101	0.1078	0.0068	0.0958	0.1006	−0.062	**0.0105**	0.701	0.372
BMI 25–29.9	B	350	219	0.1013	0.1088	0.0075	0.0959	0.1017	−0.066	**0.00777**	0.786	0.518
BMI 25–29.9	C	407	162	0.1014	0.1113	0.0099	0.0959	0.1024	−0.084	**0.00184**	0.788	0.483
BMI > 30	A	162	130	0.0839	0.0842	0.0003	0.0774	0.0809	−0.013	0.705	**0.00728**	0.505
BMI > 30	B	190	102	0.0837	0.0848	0.0011	0.0785	0.0791	−0.005	0.892	**0.0352**	0.704
BMI > 30	C	214	78	0.084	0.0841	0.0001	0.0788	0.0771	0.019	0.615	**0.043**	0.589
FF > 0.10 & < 0.2	A	665	745	0.1338	0.1368	0.0030	0.1298	0.1318	−0.032	**0.0359**	0.529	0.671
FF > 0.10 & < 0.2	B	816	594	0.1335	0.1378	0.0043	0.1293	0.1337	−0.05	**1.23E−03**	0.637	0.918
FF > 0.10 & < 0.2	C	949	461	0.1336	0.1391	0.0055	0.1292	0.1366	−0.064	**9.58E−05**	0.895	0.852
GA < 105 (days)	A	1191	1176	0.1115	0.1186	0.0071	0.1048	0.1144	−0.052	**1.38E−05**	0.405	0.0656
GA < 105 (days)	B	1437	930	0.1117	0.1201	0.0084	0.105	0.1169	−0.061	**6.38E−07**	0.96	0.395
GA < 105 (days)	C	1660	707	0.112	0.1221	0.0101	0.1056	0.1191	−0.072	**3.08E−08**	0.755	0.314

*Note:* Analyses were conducted for the full dataset and subsamples stratified by fetal sex, GA, BMI categories, and after excluding extreme FF values and IVF pregnancies. Set A (06:50 and 12:00 & 12:01 and 21:00), set B (06:50–13:00 & 13:01–21:00) and set C (06:50–14:00 & 14:01–21:00). Mann‐Whitney U *p*‐values below 0.05 are bold.

## 4 Discussion

While diurnal rhythms, governed by the body's circadian clock, regulate numerous biological processes, such as hormone secretion, metabolism, and immune function [13, 29], surprisingly little is known about whether these fluctuations extend to cfDNA dynamics. Previous studies [16, 17] have suggested that cfDNA release and clearance may fluctuate throughout the day. However, direct evidence remains limited due to a scarcity of studies, many of which are constrained by small sample sizes, methodological differences and variations in study protocols, such as differences in sampling frequency, timing and cohort composition (e.g., healthy individuals vs. cancer patients) [13, 14, 16, 17, 18, 19, 20, 21]. Here, we present the first report of intraday variation in fetal fraction (FF) in a large cohort of 2519 women pregnant with euploid single fetuses.

Our analysis revealed a significant trend: FF values were generally lower in the morning and higher in the afternoon. Multiple regression analysis demonstrated a significant positive relationship between FF and blood draw timing (06:50–21:00), even after controlling for key variables, including maternal BMI, maternal age, gestational age, and fetal sex (Figure 1; Table 2). Morning versus afternoon comparisons highlighted a mean FF difference of approximately 0.01, representing up to 10% higher FF values in the afternoon. This effect was most pronounced when dividing the dataset into the set C morning (06:50–14:00) and afternoon (14:01–21:00) groups (Figure 1; Table 3). Subgroup and sensitivity analyses confirmed the robustness of these findings across fetal sex, gestational ages, and BMI categories, as well as after excluding extreme FF values (FF > 0.1 & < 0.02) and IVF pregnancies. Notably, standardized regression coefficients (SRCs) revealed that the clock hour of blood draw (SRC = 0.139) was a stronger predictor of FF than gestational age (SRC = 0.110) or fetal sex (SRC = −0.062), second only to BMI (SRC = −0.376). This unexpected finding may be partly due to our cohort composition, as 94% of pregnancies were between gestational weeks 10 and 15 (Table 3, Table S1), a period when FF increases slowly compared to later stages (≥ 21 weeks [5, 6, 7]). Thus, in early pregnancy, diurnal variation in blood draw timing may have a stronger effect on FF than modest gestational age‐related changes. Similarly, while FF was only ∼0.003 higher in female versus male fetuses (Table 2), intraday FF differences reached ∼0.01 (Table 3), suggesting that diurnal timing may overshadow or be comparable to fetal sex effects (cf. Forgacova et al. [5]). These findings underscore the need to consider blood draw timing in both research and clinical FF assessments.

To assess the clinical significance of these findings, it is essential to evaluate the magnitude of intraday FF variation in the context of NIPT performance. While a mean FF difference of 0.01 between morning and afternoon samples may seem small relative to the typical range of 0.1–0.15 between gestational weeks 10 and 20, it could be clinically relevant for borderline cases near assay‐specific FF thresholds. For instance, 1%–8% of pregnant women fail to meet NIPT assay‐specific FF thresholds (typically 0.02–0.04), leading to screening failures [5, 7, 9]. Current strategies, such as redrawing blood 2 weeks after an initial failure, achieve only limited success, with 56%–66% of repeat NIPT tests yielding valid results [1, 9, 10]. Given that diurnal variation is associated with higher FF values in the afternoon, cases with FF values just below the threshold in the morning might, in some instances, cross into the reportable range later in the day. Likewise, higher FF levels generally enhance the statistical discrimination between euploid and aneuploid pregnancies, reducing the likelihood of false‐positive and false‐negative results [9, 30, 31]. Diurnal FF variation thus provides a biologically plausible rationale for exploring timing adjustments in blood draw protocols. Routinely scheduling NIPT tests later in the day or conducting redraws in the afternoon could potentially improve assay success rates, particularly for borderline FF cases. Given the low‐risk and practical nature of timing interventions, prospective studies are needed to confirm their effectiveness and explore their clinical impact. Shifting NIPT blood draws in the afternoon may present likely manageable logistical challenges including clinic workflow, patient scheduling, and lab processing times. Notably, approximately 50% of our samples were already collected in the afternoon, suggesting that gradual implementation would likely not significantly disrupt existing practices. As a practical interim approach, afternoon blood draws could be selectively prioritized for cases at higher risk of test failure and redraws could be scheduled in the afternoon 2 weeks after an initial failure until prospective studies provide clearer guidance. If validated, optimizing blood draw timing could represent a simple yet impactful refinement of NIPT protocols, improving test performance and outcomes for clinicians and patients alike.

While the clinical implications of diurnal FF variation are promising, understanding the underlying biological mechanisms is equally crucial for advancing NIPT protocols. What drives the observed trend of intraday FF variation between morning and afternoon blood draws? Numerous factors may influence cfDNA levels in maternal plasma, including maternal activity, dietary intake, inflammation, and circadian rhythms [6, 13, 14]. Circadian regulation of cfDNA release and/or clearance may provide a plausible explanation, as these rhythms influence many physiological processes [13, 31]. However, further research is needed to confirm this hypothesis and identify other potential contributors, such as placental physiology or maternal metabolic states. To fully elucidate the mechanisms behind intraday FF variation, fine‐scale studies are required. Longitudinal sampling of the same individuals at multiple time points throughout the day would allow researchers to track temporal dynamics more precisely. Additionally, studies investigating maternal and placental physiology could provide insight into how these factors interact to influence FF.

In summary, our discovery of diurnal variation in FF adds a new dimension to the understanding of cfDNA dynamics, emphasizing the importance of timing in both research and clinical practice. Refining protocols to account for these temporal effects could enhance the accuracy and accessibility of cfDNA‐based diagnostics, particularly in prenatal care.

## 5 Conclusions

This study is the first to demonstrate diurnal variation in fetal fraction (FF) within a large cohort of pregnant women, revealing a consistent trend of lower FF values in the morning and higher values in the afternoon. While the observed variation (approximately 10% on average) is subtle, it represents a significant addition to our understanding of FF dynamics and highlights the influence of temporal factors alongside established predictors such as BMI, gestational age, and fetal sex. These findings suggest that the timing of blood draws may meaningfully impact FF levels, offering practical implications for noninvasive prenatal testing (NIPT), particularly for women at risk of failing assay‐specific FF thresholds. By favoring afternoon blood draws or scheduling redraws later in the day, healthcare providers could potentially reduce screening failures, minimize the need for repeat testing, and shorten diagnostic delays. The mechanisms driving this intraday variation remain uncertain but are likely influenced by maternal circadian rhythms, metabolic activity, and possibly postprandial effects. Further research is needed to clarify these processes through fine‐scaled longitudinal studies of FF levels within individuals at multiple time points, coupled with investigations into maternal and placental physiology. If confirmed by prospective studies, timing optimization of blood draws could represent a simple, low‐risk intervention to enhance NIPT precision, efficiency, and accessibility. These advancements have the potential to improve clinical outcomes, reduce healthcare burdens, and ensure better experiences for expectant parents and clinicians alike.

## Ethics Statement

The authors have nothing to report.

## Consent

Informed consent was obtained from all individuals included in this study or their legal guardians or wards.

## Conflicts of Interest

The authors declare no conflicts of interest.

## Supporting information

Figure S1

Table S1

## Data Availability

The data that supports the findings of this study are available in the Supporting Information of this article.
