# Supplementary material for: Diurnal Effects on the Fraction of Fetal Cell‐Free DNA in Maternal Plasma
Source: Prenat Diagn. 2025 Jun 18;45(8):979–87. doi: 10.1002/pd.6836 (PMC12254438; doi:10.1002/pd.6836)
Supplement: Supplementary file 1 — Figure S1 [file PD-45-979-s001.docx]

**Supplemental material**

**
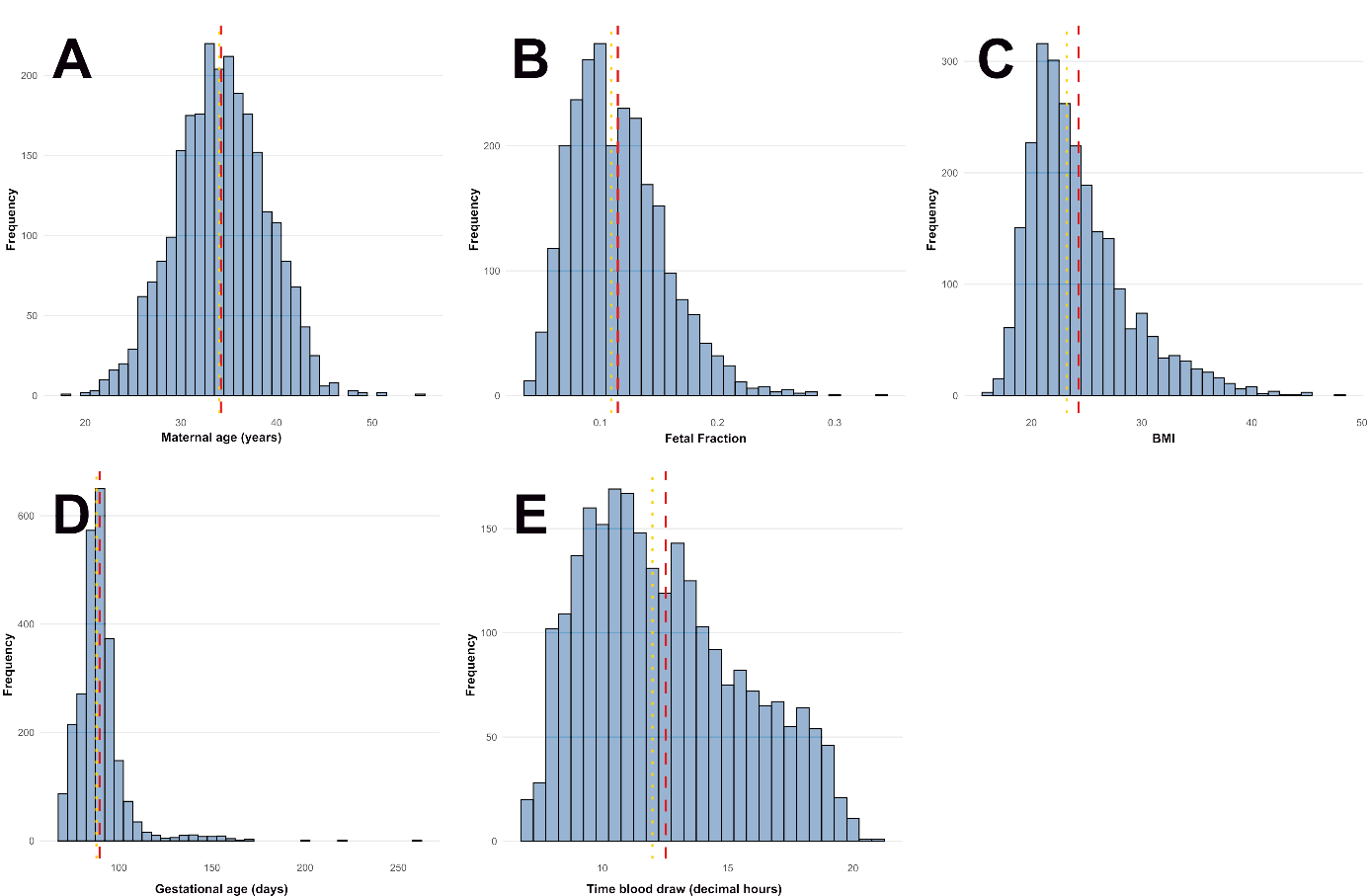
**

**Supplementary Figure S1:** Frequency histograms of basic characteristics of the study population. (A) Maternal age in years (mean: 34.18; median: 34), (B) fetal fraction (mean: 0.12 ; median: 0.11), (C) Maternal BMI (kg/m^2^) (mean: 24.28 ; median: 23.18), (D) Gestational age (days) (mean: 89.69 ; median: 88), (E) Time blood draw (decimal hours) (mean: 12.54 ; median: 12). Vertical orange dotted line indicates mean and vertical red dashed line indicates median values, respectively.

**Supplementary File 1.** Aggregated dataset of the of the study population of 2519 pregnant women, including maternal age, body mass index (BMI), gestational age, FF, time of blood draw, fetal sex, and IVF status.
